# Barriers and facilitators for the implementation of an integrated evaluation for older adults including a digital cognitive tool in primary care settings in Mexico City

**DOI:** 10.1002/alz70860_099405

**Published:** 2025-12-23

**Authors:** Mariana López‐Ortega

**Affiliations:** ^1^ Instituto Nacional de Geriatría (National Institute of Geriatric), Mexico City, DF, Mexico

## Abstract

**Background:**

In Mexico, cognitive impairment and dementia go undetected due to lack of policies and complementary services. In addition, health care professionals (HCPs) are rarely trained in geriatrics/gerontology, the diagnosis and management of these conditions. Lack of training not only prevents optimal care, but generates important barriers such as misconceptions and that hinder health care services.

**Methods:**

As part of the Davos Alzheimer's Collaborative System Preparedness (DAC‐SP) Early Detection program, a pilot was implemented in four primary care clinics in Mexico City's public health services. 16 primary care HCPs participated. They underwent pre‐program training sessions, completed two tests to assess their knowledge, confidence, attitudes, and behaviors towards dementia care in their daily practice (baseline and end of pilot). Semi‐structured interviews were conducted to further investigate these topics. Participants 60 years+ were recruited during regular clinic visits. They were evaluated using the World Health Organization's Integrated Care for Older People Assessment (ICOPE) and a digital cognitive assessment tool (DCA). Participants with DCA indicating impairment were offered a blood biomarker test.

Guided by the Consolidated Framework of implementation Research (CFIR), interviews were designed and conducted to explore the topics of interest and the process of implementation.

**Results:**

Survey results showed low confidence of HCPs in providing dementia care (diagnosis to management). Main barriers reported by HCPs were: insufficient time with patients (70%), lack of knowledge on referral of patients for diagnosis/specialist (40%), and high costs of screening, diagnosis and management (50%). Preliminary analysis of the interviews shows an increase in awareness of the of cognitive assessment as fundamental part of older adult's primary care and positive affect of training received in their daily practice. However, HCPs also reported how increased knowledge assessment instruments increase awareness of the barriers faced in the diagnosis‐management of cognitive impairment/dementia. Inner and outer settings also presented challenges to implementation, mainly, compatibility with workflow, system, processes, and perceived lack of support from Directives.

**Conclusion:**

HCPs show overall acceptance of innovative tools for comprehensive assessment of older adults, however, relevant inner and outer setting barriers need to be eliminated for these to be integrated in to usual primary care.

